# Fatty acids-based compounds as novel prophylaxis or treatment for oropharyngeal *Neisseria gonorrhoeae:* an *in vitro* study

**DOI:** 10.1080/22221751.2026.2678644

**Published:** 2026-05-22

**Authors:** Syed Ameer Hamza, Rita Paolini, Caroline Moore, Magnus Unemo, Tami Yap, Michael McCullough, Alyce Mayfosh, Thomas Rau, Stephen Kleid, Jane S. Hocking, Fabian Ys Kong, Antonio Celentano

**Affiliations:** a Melbourne Dental School, The University of Melbourne, Carlton, VIC, Australia; b WHO Collaborating Centre for Gonorrhoea and other STIs, Örebro University, Örebro, Sweden; c Institute for Global Health, University College London, London, UK; d Wintermute Biomedical Inc., Missoula, MT, USA; e Masada Private Hospital, Ramsay Health, St Kilda East, VIC, Australia; f Centre for Epidemiology and Biostatistics, Melbourne School of Population and Global Health, The University of Melbourne, Carlton, VIC, Australia

**Keywords:** Oropharyngeal gonorrhoea, *Neisseria gonorrhoeae*, antimicrobial resistance, fatty acid antimicrobials, bacterial infections

## Abstract

Oropharyngeal gonorrhoea remains a global challenge due to rising antimicrobial resistance and limited therapeutic options, necessitating urgent calls for new treatments and prophylaxis. This study evaluates the in vitro activity of arginine-based fatty acids against *Neisseria gonorrhoeae* (NG) strains in a validated oropharyngeal model. Five oropharyngeal cell lines (tonsillar/buccal/gingival/floor of mouth/uvular) were infected with two NG strains. Intracellular infection was assessed at 15/30/60 min. MIC₉₀ and cytotoxicity of five arginine-based fatty acids were determined. Promising candidates were evaluated for NG clearance at 30/60/120 min (post-infection) and prevention of infection (pre-infection) after 30/120 min drug exposure. Arginine-undecanoate (AU) and arginine-laurate (AL) were the most effective fatty-acids, showing potent bactericidal activity, with no cytotoxicity in any cell lines up to 300 µg/mL. In post-exposure experiments, AL at 150 µg/mL and at MICx3 achieved rapid, dose-dependent clearance of ≥94% for FA1090 and ≥90% for WHO R respectively within 30 min in all cell lines except in uvular cells. AU showed time-dependent killing with clearance of ≥95% for FA1090 and ≥88% for WHO-R at 120 min at 150 and 108 µg/mL (MICx3) respectively. In pre-exposure experiments, AL at maximum non-cytotoxic concentration (300 µg/mL) was more effective than AU, achieving 83·3-97·4% clearance in all cell lines within 30 min. AL and AU can be promising, non-toxic candidates for the prevention or treatment of oropharyngeal NG. Use, pre- and post-sex via topical formulations, could potentially reduce oropharyngeal NG transmission.

## Introduction

Gonorrhoea, a sexually transmitted infection caused by *Neisseria gonorrhoeae* (NG), represents a significant public health concern, with at least 82 million new cases among adolescents and adults annually [1]. Rapid emergence of antimicrobial resistance (AMR) in NG to all antibiotic classes, including last-line treatment ceftriaxone, further limits infection management [2]. While emerging therapies such as zoliflodacin and gepotidacin show promise for urogenital gonorrhoea, cure rates in oropharyngeal infections remain below the World Health Organization (WHO)’s 95% efficacy threshold [3, 4].

The oropharynx plays a central role in both treatment failure and the development of AMR. Oropharyngeal infections are often asymptomatic, complicating detection and timely treatment, and systemic antibiotics may achieve suboptimal concentrations at the oropharyngeal site [5]. Moreover, commensal bacterial species in the oropharynx facilitate AMR via horizontal gene transfer [6].

Current preventive strategies have proven ineffective: condoms are limited by low compliance [7], commercial mouthwashes have shown no benefit in clinical trials [8] and interventions such as doxycycline post-exposure prophylaxis (doxyPEP) [9] and vaccination using meningococcal (4CMenB) vaccines [10] have also failed to control oropharyngeal infections. These challenges highlight the urgent need for novel approaches to control oropharyngeal infections. One novel and innovative strategy is the use of topical oral products administered before or after oral sex to prevent incident oropharyngeal infections. NG primarily infects superficial epithelial tissue [11], making topical interventions – such as chewing gums or sprays – potentially more effective than systemic drugs, which face pharmacokinetic barriers in achieving adequate concentrations at the oropharyngeal epithelium [5]. Moreover, oropharyngeal NG infections have lower bacterial loads than urogenital or anorectal infections [12], suggesting that lower drug concentrations may suffice for prevention. Lastly, many oropharyngeal NG will have been naturally cleared within three months without treatment [13] and missed during routine 3-monthly STI screening. Therefore, intervening at the time of oral sex is one possible intervention to prevent ongoing transmission.

Fatty acids have potent antimicrobial properties that are gaining attention for their therapeutic potential [14]. Medium- to long-chain fatty acids disrupt bacterial membranes by embedding in lipid bilayers, increasing permeability and causing lysis, or impairing energy metabolism and enzyme function, diminishing bacterial viability [15]. Fatty acids have additional advantages over conventional antibiotics with low resistance development potential due to their non-specific and dynamic, structural modes of action [15, 16]. Lauric acid exhibits strong bactericidal and antiviral properties [17], while undecanoic acid also exhibits antifungal properties [18]. Fatty acids suffer from low aqueous solubility but combining them with L-arginine can improve solubility without compromising antimicrobial potency [19]. Fatty acids are well-suited for mucosal applications, as they are naturally present in saliva, and are classified as Generally Recognized as Safe (GRAS) by the US Food and Drug Administration [19]. Lastly, as fatty acids are found naturally in human diets, marketing authorization of a final therapeutic product as a food or vitamin rather than a medicine, can accelerate market access. These properties make them attractive candidates for incorporation into portable mucosal delivery systems such as chewing gums and sprays, allowing for direct exposure at the site of infection.

In this study, we investigated the *in vitro* activity and cellular toxicity of five arginine-based fatty acid compounds and the two most promising compounds (arginine-undecanoate (AU) and arginine-laurate (AL)) were tested against clinically relevant NG strains (FA1090 and WHO R/FC428) using our validated *in vitro* 2D human oropharyngeal infection model of NG [20]. This was performed to identify promising candidates for the prevention or treatment of oropharyngeal gonorrhoea, and to our best knowledge, this is the first *in vitro* study evaluating fatty acid derivatives for prevention or treatment of oropharyngeal gonorrhoea, addressing a critical unmet need in gonorrhoea control.

## Materials and methods

Briefly, we started with initial screening of five compounds using the three cell lines we used in our validated *in vitro* 2D human oropharyngeal infection model of NG [20]. After that, we expanded our *in vitro* oropharyngeal infection model [20] with addition of two epithelial cell lines (buccal and uvular). Thus, in total, five human oropharyngeal epithelial cell lines (tonsillar, buccal, gingival, floor of mouth (FOM), uvular) were infected/co-cultured (co-culture supplementary methods) with two strains of NG (antibiotic-susceptible FA1090 and multidrug-resistant WHO R/FC428). Infected cells were treated with AU and AL to assess intracellular clearance (post-exposure experiments). In separate experiments, uninfected cells were pre-exposed to AU or AL and then challenged with NG (pre-exposure experiments). Extracellular bacteria were eliminated with gentamicin prior to cell lysis, thus, colony-forming units (CFUs) reflect intracellular bacteria only. After lysis, intracellular bacteria were quantified by plating cell lysates and, after incubation, counting CFUs with an automated colony counter (Scan1200™, Interscience, France).

### Fatty acid-based compounds

Five fatty acid–based compounds (AU, AL, undecylenate (undecyclenic acid), linoleate (linoleic acid), and monodecanoate (decanoic acid)) were screened for efficacy (MIC_90_) and cytoxicity. All compounds were supplied by Wintermute Biomedical (https://www.wintermutebiomedical.com).

### Oral epithelial cell lines and culture conditions

Five epithelial cell lines (tonsillar, buccal, gingival, FOM, and uvular) representing the anatomical variation across the human oral and oropharyngeal axis (supplementary Figure 1) were used. Their characteristics and culturing conditions are summarized in supplementary Table 1. Uvular cells are not commercially available, so we isolated and immortalized primary human uvular cells in our lab. Isolation and immortalization protocols are available in supplementary methods.

### Ethics approval

This study was conducted in accordance with the ethical principles outlined in the Declaration of Helsinki. This project was approved by University of Melbourne Human Research Ethics Committee (approval 2023-27876-47484-3), the Masada Ramsay National Research Unit (2023/PID/0213; 2023/RGO/0240). Written informed consent was obtained from the donors of primary human uvular cells before their participation in the study.

### Minimum inhibitory concentration (MIC_90_)

MIC_90_ values (mg/L) for the fatty acids were determined by microbroth dilution (MBD) as previously described [20]. Briefly, bacterial suspensions (OD600 = 1.0 in PBS) were diluted 1:25 to obtain working stocks. Fatty acids were serially diluted using modified Fastidious Broth (mFB) in 96-well plates (Corning, 3370), and 100 µL of inoculum was added to each well. Plates were sealed with gas-permeable film (Merck, BR701364) and incubated for 24 h using a NIVO plate reader (Revity Group) with continuous shaking (300 rpm), and optical density (600 nm) was recorded hourly. MIC_90_ was defined as the lowest concentration inhibiting ≥90% bacterial growth as shown in (Table 1). Two reference strains (WHO F and ATCC 49226) were also used for quality control. MIC values were further validated using agar dilution according to WHO methodology.

### Cytotoxicity assay

Cytotoxicity was assessed by lactate dehydrogenase (LDH) release. Oropharyngeal epithelial cells were seeded in 48-well plates (CLS-3338, Corning®, Sigma-Aldrich, Australia) in triplicate (uvular, gingival, and FOM: 1 × 10⁴ cells/well; tonsillar and buccal: 2 × 10⁴ cells/well). Once 40–60% confluent (approximately 6 × 10⁴ cells/well), cells were treated with fatty acids (50–500 µg/mL to determine maximal non-cytotoxic concentrations) or at 1×, 2×, and 3× MIC (to determine therapeutic concentrations) for 0·5, 1, 2, or 4 h. Forty-five minutes before each endpoint, a lysis buffer was added to one replicate to serve as the maximum lysis control. After incubation, 50 µL of supernatant was transferred to a 96-well plate, followed by 50 µL LDH reagent, 30 min incubation at 37°C, and addition of 50 µL stop solution. Absorbance was read at 490 nm using a Biotek 800 TS microplate reader (BioTek, Currumbin, QLD, Australia).

### Microscopy visualization of NG in oropharyngeal cells

**Figure 1.**
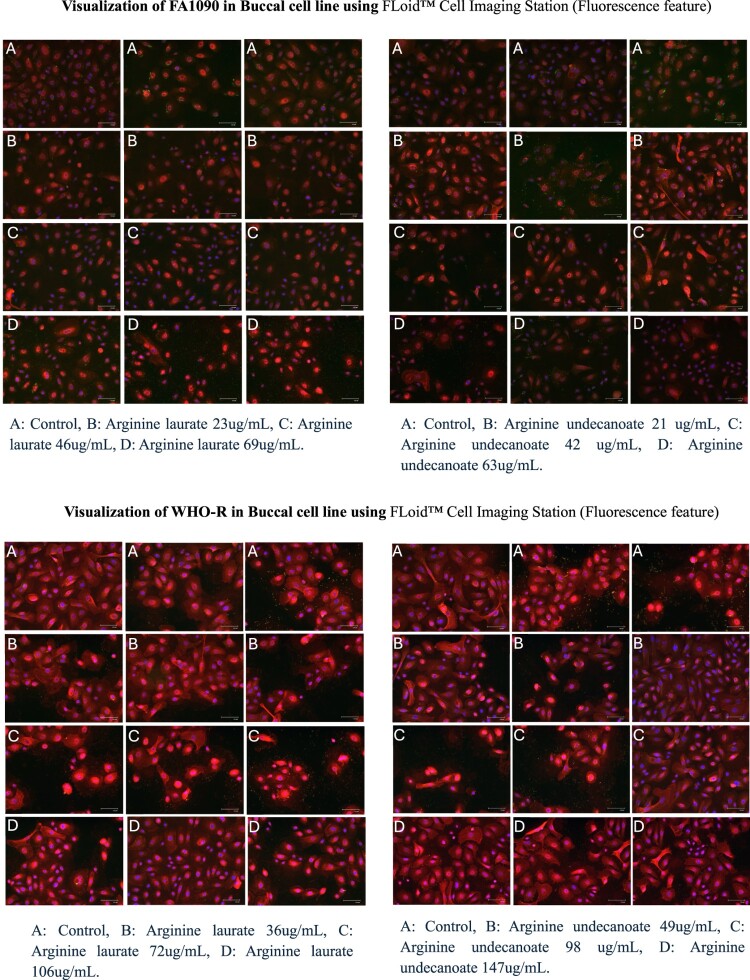
Fluorescence microscopy confirming *Neisseria gonorrhoeae* invasion of buccal mucosa cells. Epithelial cells were infected with *N. gonorrhoeae* (FA1090 and WHO-R strains) and treated with arginine-undecanoate (AU) and arginine-laurate (AL) at 1×, 2×, and 3× MIC. Cells were stained for membranes (WGA–Alexa Fluor 594, red), nuclei (DAPI, blue), and gonococci (NGab–FITC, green). Representative 3× images (AAA, BBB, etc.) were acquired using the Invitrogen™ FLoid™ Cell Imaging Station. Bright-field images were captured at 80× magnification with 21% brightness, and corresponding fluorescence images were captured at 80× magnification with 40% brightness.

Oropharyngeal epithelial cells (1 × 10⁵ cells/well) were cultured in chamber slides (#54526, Thermo Fisher Scientific, Australia) and infected as previously described [20]. Briefly, following infection, cells were treated with fatty acid–based compounds (AU, AL), then fixed with 4% paraformaldehyde. Cells were blocked with 1% BSA and subjected to fluorescence staining using WGA–Alexa Fluor 594, FITC-conjugated N. gonorrhoeae antibody, and DAPI. Slides were mounted and imaged using an Invitrogen™ FLoid™ Cell Imaging Station at 80× magnification. Representative images of buccal cells under control and AU/AL treatments are shown in Figure 1.

### Statistical analysis

Statistical analyses were performed using GraphPad Prism version 10·6·0 (GraphPad Software, Boston, USA). Each experiment was conducted in technical triplicate, and data are presented as mean ± standard deviation. For intracellular CFU assays, results were normalized to untreated controls and analyzed using two-way ANOVA to assess the effects of pre-exposure (30 and 120 min at 300 μg) and post-exposure (30, 60, and 120 min) treatments at different concentrations. Cytotoxicity data were analyzed by one-way ANOVA, normalized to maximum lysis, and compared to untreated controls.

### Role of the funding source

The funders had no role in study design, data collection, analysis, interpretation, or writing of the report of this study.

## Results

### Initial cytotoxicity screening and MICs of fatty acids

Five fatty acid–based compounds were initially screened across three oropharyngeal epithelial cell lines (gingival, tonsillar, and floor-of-mouth) to assess cytotoxicity (Supplementary Table 3), and their minimum inhibitory concentrations (MICs) were determined (Table 1). While all five compounds exhibited antimicrobial activity, three demonstrated unacceptable cytotoxicity toward host cells and were therefore excluded. In contrast, arginine laurate (AL, C12) and arginine undecanoate (AU, C11), both medium-chain fatty acid derivatives, showed no detectable cytotoxicity across all tested cell lines while retaining antimicrobial activity. These compounds were therefore selected for further evaluation against Neisseria gonorrhoeae strains in the 2D human oropharyngeal infection model, as they provided the most favourable balance between efficacy and host cell compatibility.

**Table 1.** MIC_90_ values (mg/L) using microbroth and agar dilution assay. *Final compounds tested in the 2D human oropharyngeal infection model [20].

Fatty acids (mg/L)	Agar (or microbroth) dilution MIC (mg/L)			
	FA1090	WHO R	WHO F (control)	ATCC 49226 (control)
Arginine undecanoate * (AU, undecanoic acid)	16 (21)	64 (36)	32	64
Arginine laurate * (AL, lauric acid)	32 (23)	32 (49)	32	32
Arginine-linoleate (linoleic acid)	Not tested (>50)	Not tested (33)	Not tested	Not tested
Arginine undecylenate (undecylenic acid)	32 (46)	128 (31)	64	128
Arginine monodecanoate (decanoic acid)	64 (48)	128 (31)	64	64

### Cytotoxic assessment

**Figure 2.**
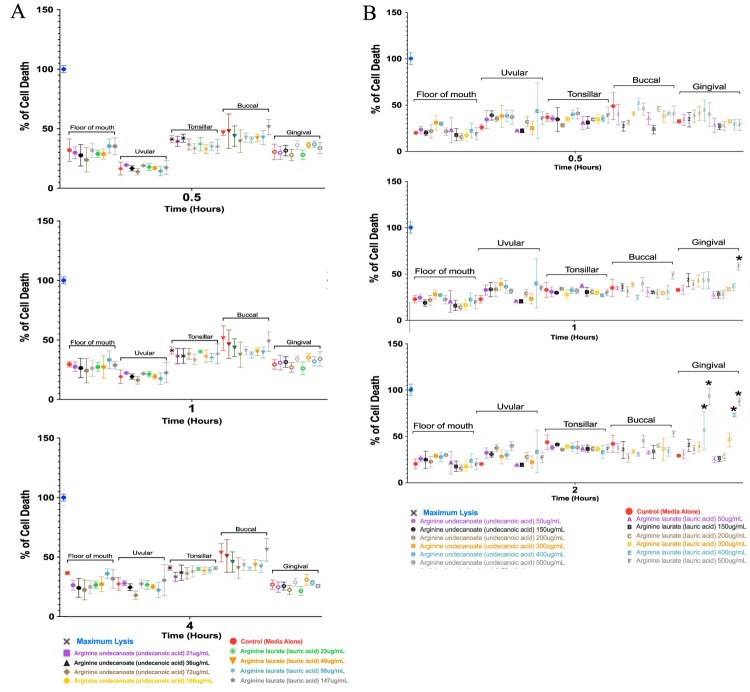
(A): Cytotoxicity of arginine-undecanoate (AU) and arginine-laurate (AL) at MIC 1, 2, and 3. (B): Cytotoxicity of AU and AL at 50–500 μg/mL. Data are normalized to maximum lysis (blue) and presented as mean ± SD. Statistical significance (*p* < 0·05) is indicated as follows: *compared to untreated control, # indicates dose-dependent effect, and ^ indicates time-dependent effect.

The cytotoxicity of AU and AL at 1–3× MIC was assessed across five oropharyngeal cell lines. As shown in Figure 2A, neither compound induced substantial cytotoxicity in any cell line. Based on these results, an additional concentration (150 µg/mL) was tested for FA1090 to align with the 147 µg/mL concentration used for WHO R. To define the upper safe limit for pre-exposure, a broader concentration range (50–500 µg/mL) was evaluated. AU and AL were non-cytotoxic up to 300 µg/mL at all time points; however, higher concentrations (400–500 µg/mL) induced significant cytotoxicity exclusively in gingival cells after 1 h (Figure 2(B)). Accordingly, 300 µg/mL was selected for subsequent pre-exposure experiments.

#### Ph of culture media

The pH of the culture media containing fatty acid compounds remained within physiological ranges, indicating that these compounds did not disrupt the oropharyngeal epithelial cell environment (supplementary Table 2).

### Assessment of bacterial clearance by applying compounds before and after infecting oropharyngeal cells with *Neisseria gonorrhoeae*:

#### *Neisseria* invasion into oropharyngeal epithelial cells

The five oropharyngeal epithelial cell lines (tonsillar, buccal, gingival, FOM, uvular) were infected with NG strains (FA1090 or WHO R) and commensal *N. oralis* as a negative control.

**Figure 3.**
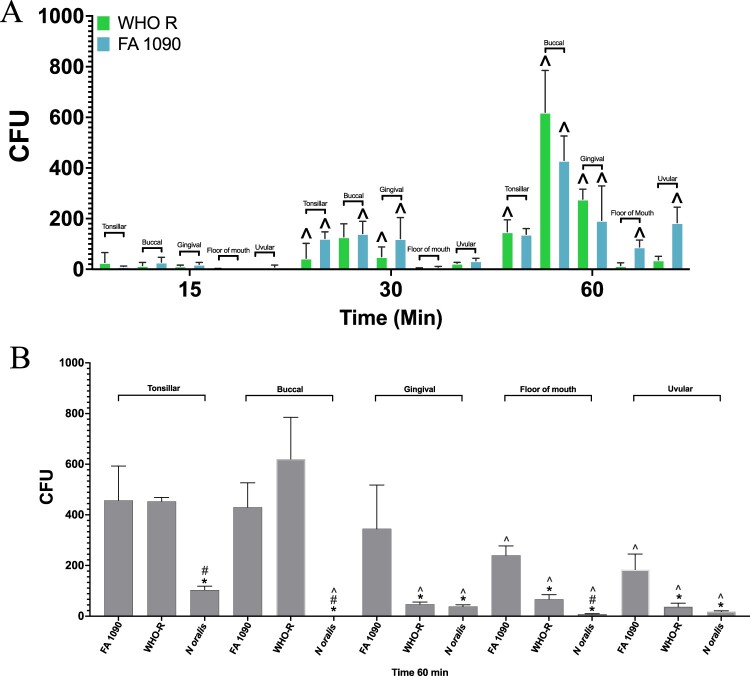
(A): Infection rate of *N. gonorrhoeae* strains (FA1090, WHO-R) across tonsillar, buccal, gingival, floor of the mouth (FOM) and uvular cell lines. ^ compared to the time dependent trend. (B): Infection rate of *N. gonorrhoeae* strains (FA1090, WHO-R) and commensal *N. oralis* across tonsillar, buccal, gingival, floor of the mouth (FOM) and uvular cell lines. Data presented as mean + SD. Statistical significance (*p* < 0·05) is indicated as follows: * compared to FA1090 within same cell line; # compared to WHO-R within same cell line; ^ compared to the same *N. gonorrhoeae* strain across different cell lines.

At 15 min, FA1090 infected all cell lines (Figure 3(A)). Highest invasion was seen in buccal (28 CFU), tonsillar (11 CFU), and gingival (10 CFU) cells, and lowest in FOM (1 CFU) and uvular (1 CFU) cells. Similar results were seen for WHO R at 15 min with highest invasion in tonsillar (27 CFU), gingival (20 CFU), and buccal (15 CFU) cells, but no invasion in FOM and uvular cells.

At 30 min (Figure 3(A)), invasion increased significantly in a time-dependent manner (*p* < 0·05) for FA1090 in buccal (141 CFU), tonsillar (122 CFU), and gingival (121 CFU) cells, while for WHO R only in buccal cells (128 CFU; *p* < 0·05). Invasion in FOM and uvular cells remained low for both NG strains (FA1090: 10 and 23 CFU; WHO R: 5 and 30 CFU) respectively.

At 60 min (Figure 3(B)), FA1090 invasion peaked in tonsillar (457 CFU) and buccal (430 CFU) cells, followed by gingival cells (345 CFU, *p* < 0·05). Invasion in FOM (240 CFU, *p* < 0·05) and uvular cells (184 CFU, *p* < 0·05) was significantly lower. Similarly, WHO R invaded tonsillar (453 CFU) and buccal (620 CFU) cells significantly higher, while gingival (47 CFU, *p* < 0·05), FOM (67 CFU, *p* < 0·05), and uvular (37 CFU, *p* < 0·05) cells showed significantly lower invasion.

For *N*. *oralis*, at 60 min invasion was minimal compared to NG strains. Tonsillar cells exhibited the highest invasion (103 CFU), followed by gingival (38 CFU, *p* < 0·05) and uvular (18 CFU, *p* < 0·05) cells. FOM (8 CFU, *p* < 0·05) and buccal (1 CFU, *p* < 0·05) cells showed negligible invasion.

### Post-exposure clearance of *Neisseria gonorrhoeae* strains (FA1090 and WHO R) in oropharyngeal cells by fatty acids:

#### Clearance of FA1090 infected cells (post-exposure)

**Figure 4.**
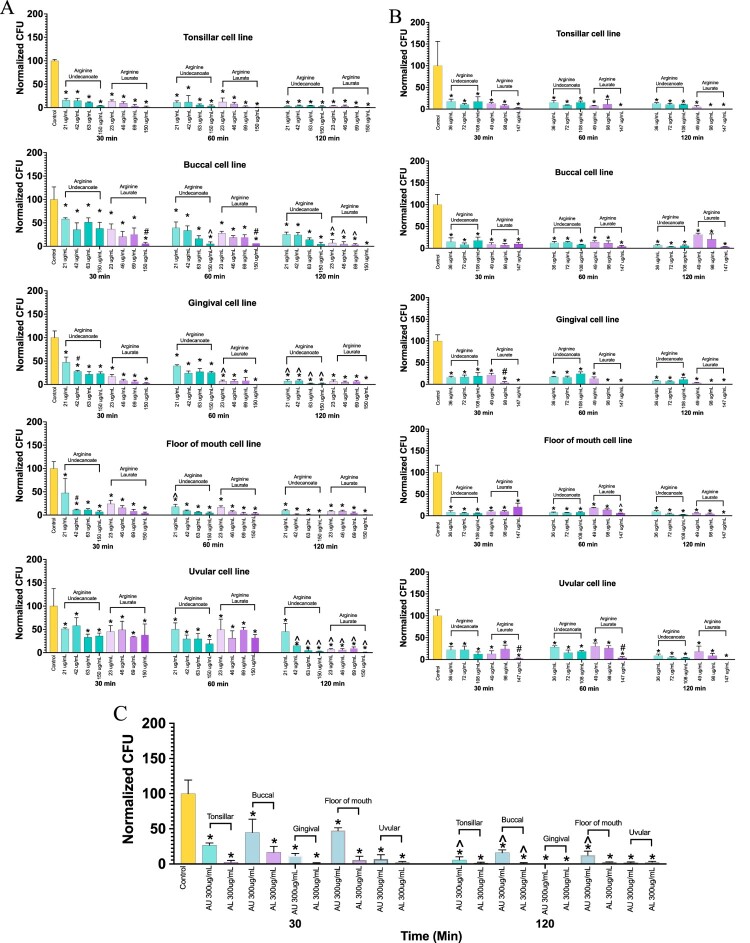
(A): Clearance of FA1090 in oropharyngeal cells using arginine-undecanoate (AU) and arginine-laurate (AL) at MIC 1, 2, 3 and 150 ug/mL; (B): Clearance of WHO-R in oropharyngeal cells using AU and AL at MIC 1, 2, and 3; C: Pre-exposure clearance of FA1090 in oropharyngeal cells using AU and AL at MIC 300 µg/mL. Data are normalized and presented as mean ± SD. Statistical significance (*p* < 0·05) is denoted as follows: *compared to control, # indicates dose dependence, and ^ indicates time dependence.

Overall, among FA1090 infected cells, AL exhibited the most effective antimicrobial activity, demonstrating both time- and dose-dependent killing, whereas AU showed time-dependent activity only (Figure 4(A); supplementary table 4A).

For AL, at the highest concentration (150 µg/mL), early bacterial clearance at 30 min of >94% was seen in all cell lines except the uvular – tonsillar (98·3% clearance), gingival (97.0%), FOM (95·6%) and buccal (94·1%) cells and lowest in uvular cells (61·8%) as compared to control. Similar results were seen at 60 min, however at 120 min, AL cleared >99% of FA1090 from all cell lines.

In contrast, for AU at 150 µg/ml, early clearance at 30 min of ≥93% was only seen in tonsillar and FOM cells (95·8% and 93·0% respectively). Similar results were seen at 60 min but with the addition of high clearance in buccal cells (94·6%). At 120 min, AU cleared >95% of FA1090 from all cell lines.

#### Clearance of WHO R infected cells (post-exposure)

Overall, and similar to FA1090-infected cells, AL displayed the most effective activity against WHO R, showing both time- and dose-dependent killing, while AU was time-dependent only (Figure 4(B); supplementary table 4B).

For AL at the highest concentration (147 µg/mL), early clearance at 30 min of >97% was observed in gingival (100%), tonsillar (97·8%), and uvular (97.7%) cells, and lowest in buccal (90%) and FOM (79·3%) cells, compared with the control. At 60 min, clearance of ≥94% was observed in all cells (100% for tonsillar and gingival), while at 120 min, AL cleared >99% of WHO R from all cell lines except buccal (96·7%).

For AU at 108 µg/mL (MIC x 3) and 30 min, higher clearance was observed only in FOM cells (94·1%), while all other cell lines showed <88% clearance. At 60 min, the results remained similar across all cell types, except for the addition of higher clearance in buccal cells (92·0%). At 120 min, AU cleared >94% of WHO R only from FOM (97.1%), uvular (96·0%) and buccal (94·2%) cells, while clearance was lower for tonsillar (89·3%) and gingival (88·8%) cells.

#### Clearance of *Neisseria gonorrhoeae* (FA1090) in oropharyngeal cells pre-treated with fatty acids (pre-exposure)

Similar to the above experiments, AL exhibited the most effective antimicrobial activity.

In experiments using the highest non-cytotoxic concentration of 300 µg/mL of AL, early clearance at 30 min of >94% was seen in all cell lines except for the buccal cells, i.e. gingival (99·2% clearance), tonsillar (97·4%), uvular (97·4%), FOM (94.4%) and for buccal (83·3%) cells compared to control (Figure 4(C); supplementary Table 4C). At 120 min, AL cleared >97% of FA1090 from all cell lines.

In contrast, AU at 300 µg/mL, early clearance at 30 min was only seen in uvular cells (93·3%) and lower in gingival (88·3%), tonsillar (73·3%), buccal (55·2%) and FOM (52·5%) cells. At 120 min, AU cleared >94% in gingival (99·7%), uvular (98·3%) and tonsillar (94·3%) cells and <88% for buccal (84·1%) and FOM (87·6%) cells.

## Discussion

The results of this study demonstrate the promising antimicrobial potential of AL and AU against oropharyngeal NG. Gonorrhoea represents a major global public health challenge due to increasing AMR, including resistance to the last-line agent ceftriaxone [2], and the suboptimal efficacy in the oropharynx also of newer drugs such as zoliflodacin and gepotidacin (91·3% and 87·5% respectively) [3, 4]. Other proposed interventions, e.g. antimicrobial mouthwashes [8], doxyPEP [9], 4CMenB vaccines [10], have shown to be ineffective, underscoring the urgent need for alternative therapeutic strategies. Because many oropharyngeal NG infections are asymptomatic and many clear spontaneously within three months [13], three-monthly screening recommendations would often fail to detect and treat infections and transmission will have already occurred. A topical preventative product around the time of oral sex would therefore represents a novel intervention.

However, for a topical intervention to be effective, a clear understanding of NG colonization within the oropharynx is essential to ensure adequate exposure to the proposed fatty acids. Significant gaps remain regarding NG colonization and growth sites in the oropharynx [21].

Early studies suggested tonsillar involvement but reported inconsistent findings for other oropharyngeal regions [11] and provided limited data on intracellular invasion. Building on our prior observations in three oropharyngeal cell lines [20], the present study assessed five distinct oropharyngeal cell types and identified strain-dependent differences in invasion. FA1090 showed rapid intracellular invasion in tonsillar, buccal, and gingival cells within 15 min, with lower invasion in FOM and uvular cells. These findings challenge assumptions that the tonsils are the primary site of oropharyngeal infection [13]. They highlight the need for pharmacokinetic studies to assess drug distribution across infected sites and ensure effective exposure to maximize cure and minimize AMR emergence [5]. A proposed controlled human infection model may further clarify colonization patterns and support evaluation of new interventions [22].

Previous studies have shown that free fatty acids and monoglycerides possess antimicrobial activity against Neisseria gonorrhoeae, primarily demonstrated in simplified in vitro systems lacking host-cell components and without assessment of cytotoxicity toward human tissues [23]. In contrast, the present study evaluates arginine-conjugated fatty acid derivatives within a 2D human oropharyngeal epithelial infection model, enabling simultaneous assessment of antimicrobial efficacy and host cell compatibility. This approach provides a more physiologically relevant framework and highlights the potential of these compounds for topical application in oropharyngeal infection. Fatty acids have also been explored as potential prophylactics, including for prevention of ophthalmia neonatorum, highlighting relevance beyond genital infection [24]. Building on this rationale, we evaluated two novel arginine-conjugated fatty acid derivatives, AL and AU, designed for topical application [19]. In post-exposure experiments, both compounds showed time- and dose-dependent antimicrobial activity against both NG strains across all oropharyngeal cell types, with AL demonstrating superior efficacy against FA1090 compared with AU.

In pre-exposure experiments, mimicking topical use before oral sex, AL at the maximum non-cytotoxic concentration (300 µg/mL) cleared >94% of FA1090 across all cell lines after 30 min, with slightly lower clearance in buccal cells (83·3%). In post-exposure experiments, AL at 150 µg/mL cleared >94% of FA1090 in all cell lines except uvular cells (61·8%). Together, these data suggest that a topical regimen using AL at 300 µg/mL before and after oral sex may be optimal, assuming salivary dilution does not reduce concentrations below 150 µg/mL. Pre-exposure efficacy is further supported by the rapid intracellular invasion of FA1090 observed within 15 min in our 2D human oropharyngeal infection model.

Although pre-exposure experiments focused on the susceptible reference strain FA1090, post-exposure assays demonstrated substantial clearance of the multidrug-resistant WHO R strain (≥90% at 30 min in most cell lines, except FOM cells [79·3%]), suggesting activity against circulating resistant gonococci of growing global concern.

Regarding delivery, mucoadhesive formulations such as medicated chewing gums may offer prolonged mucosal contact and sustained local drug concentrations; however, challenges remain [25], and additional delivery approaches such as sprays may be required to ensure coverage of the posterior oropharynx [26].

Safety is another essential consideration that must be addressed for any oral prophylactic product . In our experimental setting, AL and AU showed no cell cytotoxicity at concentrations up to 300 µg/mL, with only moderate toxicity observed above 400 µg/mL, but only in gingival cells. This favorable safety profile aligns with the GRAS classification of many fatty acids and addresses a gap in previous research, which often lacked cytotoxicity assessments in mucosal epithelial cells [14, 23, 27]. Importantly, the antimicrobial effects observed in our study were not attributable to acidification, as the culture medium pH remained within physiological range. This finding is consistent with prior reports showing that fatty acids retain antimicrobial activity under buffered conditions, supporting a membrane-disruptive rather than pH-dependent mechanism [23]. Future studies are needed to examine the impacts on healthy oral microbiota. For example, the Belgium PreGo study examining the impact of Listerine Cool Mint® mouthwash on NG infection, also found a negative effect on healthy oral microbiota [28]. It is also worth highlighting that many lifestyle behaviors can also have a negative impact on oral health [29] and the use of a topical fatty acid should be placed in this context and a topical fatty acid is likely to have substantial benefits over a systemic antibiotic. On the other hand, one of the most significant advantages of fatty acid-based antimicrobials over conventional antibiotics is their low likelihood of resistance development, given their broad and multi-target membrane-disruptive activity [30]. Lastly, our pre-exposure experiments also provided some insights into the pharmacokinetics of tested fatty acids. As cells were washed before they were exposed to NG, any subsequent reduction in intracellular CFUs is likely from fatty acids within the cells, suggesting these fatty acids were able to cross the oral cell membranes. This is encouraging as the fatty acids were conjugated to arginine to increase their water solubility, which can reduce passive diffusion of these fatty acids across lipid-based cell membranes in human tissues. More studies are needed to understand if intracellular uptake was by active or passive drug transport systems or if the fatty acids block cellular uptake of NG.

The present study has limitations. Although our 2D human oropharyngeal infection model uses a normal epithelial cell line and improves upon cancer-derived models, it does not fully recapitulate *in vivo* physiology, including salivary flow, immune components, and epithelial stratification. Advanced 3D mucosal models will be important to validate these findings and inform product development.

In conclusion, AL and AU demonstrate rapid, potent, and site-specific antimicrobial activity against oropharyngeal NG, including AMR strains, with favorable cytotoxicity profiles and a low predicted risk of AMR development. Together, these findings position AL and AU as strong candidates for future topical interventions aimed at preventing or treating oropharyngeal gonorrhoea. Further translational studies, formulation development, and clinical validation are warranted.

## Supplementary Material

Supplementary Material.docx
