# Supplementary material for: Fatty acids-based compounds as novel prophylaxis or treatment for oropharyngeal Neisseria gonorrhoeae: an in vitro study
Source: Emerg Microbes Infect. 2026 May 22;15(1):2678644. doi: 10.1080/22221751.2026.2678644 (PMC13250877; doi:10.1080/22221751.2026.2678644)
Supplement: Supplementary Material.docx [file TEMI_A_2678644_SM6963.docx]

**Supplementary Material**

**Table of contents**

[**Supplementary Methods** 2](#_Toc216777440)

[**BACTERIA AND ORAL EPITHELIAL CELLS CO-CULTURE** 2](#_Toc216777441)

[**Neisseria gonorrhoeae bacterial strains** 2](#_Toc216777442)

[**Uvular cells isolation, Ethics and immortalization** 2](#_Toc216777443)

[**Ethics** 2](#_Toc216777444)

[**Isolation and immortalization of primary human oropharyngeal cells (uvular)** 2](#_Toc216777445)

[**Supplementary Table 1** 3](#_Toc216777446)

[**Supplementary Table 2** 5](#_Toc216777447)

[**Supplementary Table 3** 6](#_Toc216777448)

[**Supplementary Table 4A** 7](#_Toc216777449)

[**Supplementary Table 4B** 8](#_Toc216777450)

[**Supplementary Table 4C** 9](#_Toc216777451)

[**Supplementary Figure 1** 10](#_Toc216777452)

[**Supplementary Figure 2** 11](#_Toc216777453)

[**Supplementary Figure 3** 12](#_Toc216777454)

# **Supplementary Methods**

# **BACTERIA AND ORAL EPITHELIAL CELLS CO-CULTURE**

## **Neisseria gonorrhoeae bacterial strains**

Three well-characterised *Neisseria* strains were used: NG FA1090 and WHO R/FC428,^21,22^ and *N. oralis* (commensal species as negative control). Bacteria were maintained in mFB and harvested during exponential growth for infection assays. Inocula were prepared in tissue culture media (supplementary Table 1), and bacterial counts were verified by plating on chocolate agar supplemented with 1% IsoVitalex. Clearance of FA1090 was the focus of our experiments.

**Co-culture / infection assay**

Oropharyngeal cells were seeded in 48-well plates (CLS-3338, Corning®, Sigma-Aldrich, Australia) in triplicate: uvular, gingival, and FOM cells at 3×10⁴ cells/well, and tonsillar and buccal cells at 6×10⁴ cells/well. Upon reaching 60–80% confluence (~1×10⁵ cells/well), cells were infected with NG (FA1090 or WHO R) or *N.* *oralis* at a multiplicity of infection (MOI) of 1:15 for 1 hour. For pre-exposure treatment, oropharyngeal cells were incubated with AU and AL (300 µg/mL – the maximum non-cytotoxic concentration) for 30 or 120 minutes before infection. For post-infection treatment, cells were infected and then treated with AU or AL at 1×, 2×, or 3× their MIC_90_ for 30, 60, or 120 minutes. For FA1090, an additional concentration (150µg/mL) for AL was tested post-infection to match the concentration used for WHO R experiments. To remove extracellular bacteria, cells were washed with PBS and treated with gentamicin (500 µg/mL) for 1 hour, followed by lysis with 1% saponin for 15 minutes. Lysates were plated on agar, and intracellular CFUs were quantified.

The pH of cell culture media containing AU and AL (50-500µg/mL) was measured using a calibrated benchtop pH meter (EUTECH Instruments, Thermo Fisher Scientific, USA), calibrated with standard buffers (pH 4.0, 7.0, 10.0) (supplementary table 2) to determine if they changed the media pH.

##

## **Isolation and immortalization of primary human oropharyngeal cells (uvular)**

Fresh tissue was collected from a patient undergoing standard care pharyngeal palatoplasty at Masada Private Hospital, Ramsay Health, St Kilda East, Victoria and placed in DMEM with 10% fetal bovine serum (FBS) for transportation to lab. Samples were then washed in PBS containing 2% penicillin-streptomycin and 500 μg/mL gentamicin for 10 min, minced into ~2–3 mm pieces, and incubated overnight at 4°C in 2.4 U/mL Dispase II (7105-041, Gibco; Life Technologies™). Epidermal sheets were digested with 0.25% trypsin at 37°C for 10 min, neutralized with growth medium, filtered through a 40 μm strainer, centrifuged at 200xg, and seeded in keratinocyte serum-free medium (K-SFM) containing 2% penicillin-streptomycin. At ~60% confluence, primary cells were spin-infected with SV40 virus (4 μg/mL polybrene, 2500 rpm, 90 min, 30°C) and expanded. Successful immortalization was confirmed by doxycycline-inducible EGFP expression (GFP fluorescence, supplementary figure 2) and by PCR detection of SV40 sequences in genomic DNA (DNeasy Kit, Qiagen; GoTaq® Green Master Mix, Promega). PCR products were resolved on a 1% agarose gel in 1X TAE at 90 V. Representative SV40-specific bands were observed at passages 10 and 25 (supplementary figure 3)

# **Supplementary Table 1**

| **Characteristics and subculturing procedures of the human oral cells used in experiments** | | | | | |
| --- | --- | --- | --- | --- | --- |
| Cell line name | Origin/  Company | Product code | Anatomical site | Type | Media characteristics and subculturing procedures |
| OKF6 | Oral Health Cooperative Research Centre (OHCRC),  The University of Melbourne, Australia | OKF6 | Floor of the mouth | Immortalised normal epithelial keratinocytes | Grown to 60–80% confluence before being further subcultured. OKF6 cells were cultured using keratinocyte serum-free medium (K-SFM) (#17005-042, Thermo Fisher Scientific, Scoresby, VIC, Australia) containing 25 µg/mL bovine pituitary extract and 0.2 ng/mL human recombinant epidermal growth factor (as per manufacturer’s instructions), 0.4 mM CaCl_2_, and 1% penicillin streptomycin mixture (P4333, Sigma-Aldrich, Castle Hill, NSW, Australia). OKF6 cells were incubated at 37°C, 5% CO_2_ for 5 to 7 days to reach 80% confluency. Epithelial cells grown to 80% confluency were subsequently detached via a pre-treatment of 10 mM EDTA for 10 min, followed subsequently with incubation with a 0.25% trypsin in 1mM EDTA solution (T4049, Sigma-Aldrich, Castle Hill, NSW, Australia) for 3-5 min. |
| Phk (Uvular) | Melbourne Dental school, The University of Melbourne, Australia | Phk-05 | Uvula | Immortalised normal epithelial keratinocytes | Grown to 60–80% confluence before being further subcultured. PhK cells were cultured using keratinocyte serum-free medium (K-SFM) (#17005-042, Thermo Fisher Scientific, Scoresby, VIC, Australia) containing 25 µg/mL bovine pituitary extract and 0.2 ng/mL human recombinant epidermal growth factor (as per manufacturer’s instructions), 0.4 mM CaCl_2_, and 1% penicillin streptomycin mixture (P4333, Sigma-Aldrich, Castle Hill, NSW, Australia). OKF6 cells were incubated at 37°C, 5% CO_2_ for 5 to 7 days to reach 80% confluency. Epithelial cells grown to 80% confluency were subsequently detached via a pre-treatment of 10 mM EDTA for 10 min, followed subsequently with incubation with a 0.25% trypsin in 1mM EDTA solution (T4049, Sigma-Aldrich, Castle Hill, NSW, Australia) for 3-5 min. |
| hTERT TIGKs | ATCC, Australia | CRL-3397^TM^ | Gingiva/  alveolar process | hTERT-immortalised normal epithelial keratinocytes | Grown to 60–80% confluence before being further subcultured. Gingival cells were cultured using ATCC gingival media (ATCPCS200030, InVitro technologies, Australia) and respective growth supplements as per manufacturer’s instructions (ATCPCS200040, InVitro technologies, Australia) containing 1% penicillin streptomycin mixture (P4333, Sigma-Aldrich, Castle Hill, NSW, Australia). Gingival cells were incubated at 37°C, 5% CO_2_ for about 3-5 days to reach 80% confluency. Epithelial cells grown to 80% confluency were subsequently detached via a pre-treatment of 10 mM EDTA for 5 min, followed subsequently with incubation with a 0.25% trypsin in 1mM EDTA solution (T4049, Sigma-Aldrich, Castle Hill, NSW, Australia) for 1-3 min. |
| HTEpiC | ScienCell Research Laboratories, Wangara, Western Australia, Australia | #2560 | Tonsil | Primary normal epithelial keratinocytes | Grown to 60–80% confluence before being further subcultured. Tonsil cells were cultured using TEpiCM (2561, ScienCell) basal media and respective growth supplements as per manufacturer’s instructions (#2301, ScienCell, Wangara, Western Australia, Australia) containing 1% penicillin streptomycin mixture (P4333, Sigma-Aldrich, Castle Hill, NSW, Australia). Tonsil cells were incubated at 37°C, 5% CO_2_ for about 7 days to reach 80% confluency. Epithelial cells grown to 80% confluency were subsequently detached via a pretreatment of 10 mM EDTA for 3-5 min, followed subsequently with incubation with a 0.25% trypsin in 1mM EDTA solution (T4049, Sigma-Aldrich, Castle Hill, NSW, Australia) for 1 min. |
| Buccal | ScienCell Research Laboratories, Wangara, Western Australia, Australia | #2610 | Buccal | Primary normal epithelial keratinocytes | Grown to 60–80% confluence before being further subcultured. Buccal cells were cultured using Oral Keratinocytes medium (2611, ScienCell) basal media and respective growth supplements as per manufacturer’s instructions (#2652, ScienCell, Wangara, Western Australia, Australia) containing 1% penicillin streptomycin mixture (P4333, Sigma-Aldrich, Castle Hill, NSW, Australia). Buccal cells were incubated at 37°C, 5% CO_2_ for about 7 days to reach 80% confluency. Epithelial cells grown to 80% confluency were subsequently detached via a pretreatment of 10 mM EDTA for 3-5 min, followed subsequently with incubation with a 0.25% trypsin in 1mM EDTA solution (T4049, Sigma-Aldrich, Castle Hill, NSW, Australia) for 1 min. |

# **Supplementary Table 2**

| **pH measurements of culture media containing fatty acid-based compounds (AU and AL)** | | | | |
| --- | --- | --- | --- | --- |
|  | Tonsillar | Buccal | Floor of mouth / Uvular | Gingival |
| Arginine Laurate /  Arginine undecanoate (µg/mL) |  |  |  |  |
| 500 | 7.3/7.26 | 7.42/7.39 | 7.3/7.16 | 7.18/7.16 |
| 400 | 7.31/7.26 | 7.42/7.39 | 7.21/7.16 | 7.19/7.16 |
| 300 | 7.35/7.27 | 7.42/7.39 | 7.25/7.17 | 7.15/7.14 |
| 200 | 7.33/7.28 | 7.35/7.36 | 7.27/7.28 | 7.18/7.20 |
| 150 | 7.29/7.25 | 7.27/7.25 | 7.29/7.25 | 7.21/7.17 |
| 50 | 7.31/7.27 | 7.13/7.15 | 7.09/7.17 | 7.11/7.16 |

# **Supplementary Table 3**

| **Screening of five fatty acid for cytotoxic effects in representative oropharyngeal epithelial cell lines** | | | |
| --- | --- | --- | --- |
| Compounds μg/mL | Gingival | Floor of mouth | Buccal |
| Arginine undecanoate  (undecanoic Acid) | **-** | **-** | **-** |
| Arginine laurate  (lauric acid) | **-** | **-** | **-** |
| Arginine-linoleate  (linoleic acid) | **+** | **+** | **+** |
| Arginine undecylenate  (undecylenic acid) | **-** | **-** | **+** |
| Arginine monodecanoate  (decanoic acid) | **-** | **+** | **+** |

“+” stands for cytotoxic to oropharyngeal epithelial cell lines and “–” stands for non-cytotoxic to oropharyngeal epithelial cell lines.

# **Supplementary Table 4A**

| **Post exposure clearance of FA1090 in oropharyngeal cells using AU and AL at MIC 1,2,3 and 150µg/mL** | | | | | | | | |
| --- | --- | --- | --- | --- | --- | --- | --- | --- |
| Cell line | AU | | | | AL | | | |
| Time (30min) | | | | | | | | |
|  | MIC 1  (21 µg/mL) | MIC 2  (42 µg/mL) | MIC 3  (63 µg/mL) | MIC 7  (150 µg/mL) | MIC 1  (23 µg/mL) | MIC 2  (46 µg/mL) | MIC 3  (69 µg/mL) | MIC 7  (150 µg/mL) |
| Tonsillar | 84.0 | 85.1 | 89.4 | 95.8 | 86.3 | 90.6 | 95.9 | 98.3 |
| Buccal | 41.5 | 64.3 | 48.5 | 61.9 | 63.2 | 79.3 | 74.2 | 94.1 |
| FOM | 52.3 | 88.7 | 88.7 | 93.0 | 75.6 | 84.1 | 90.8 | 95.6 |
| Uvular | 48.7 | 41.8 | 66.7 | 64.0 | 54.5 | 50.9 | 65.7 | 61.8 |
| Gingival | 52.5 | 71.9 | 78.1 | 76.6 | 82.9 | 91.7 | 93.3 | 97.0 |
| Time (60min) | | | | | | | | |
| Tonsillar | 89.2 | 88.0 | 94.1 | 96.5 | 87.6 | 92.2 | 97.5 | 99.5 |
| Buccal | 60.8 | 65.9 | 83.6 | 94.6 | 72.1 | 81.5 | 80.6 | 93.5 |
| FOM | 82.2 | 90.6 | 93.6 | 95.1 | 83.0 | 92.5 | 96.4 | 96.3 |
| Uvular | 49.7 | 70.1 | 70.0 | 80.8 | 50.4 | 68.4 | 50.7 | 68.0 |
| Gingival | 60.2 | 75.7 | 72.6 | 74.5 | 92.8 | 92.8 | 91.3 | 100 |
| Time (120min) | | | | | | | | |
| Tonsillar | 96.6 | 95.9 | 95.3 | 97.5 | 96.3 | 96.8 | 98.6 | 99.6 |
| Buccal | 74.5 | 75.8 | 85.7 | 95.4 | 93.0 | 95.1 | 95.9 | 99.1 |
| FOM | 90.2 | 98.7 | 98.9 | 99.8 | 91.9 | 91.4 | 94.7 | 99.5 |
| Uvular | 54.6 | 84.9 | 95.3 | 96.8 | 92.9 | 94.1 | 90.4 | 99.1 |
| Gingival | 92.7 | 91.9 | 97.8 | 97.9 | 93.6 | 94.8 | 93.2 | 100 |

Green presenting ≥ 95% clearance and orange representing 90-94.9% clearance

# **Supplementary Table 4B**

| **Post exposure clearance of WHO-R in oropharyngeal cells using AU and AL at MIC 1,2, and 3** | | | | | | |
| --- | --- | --- | --- | --- | --- | --- |
| Cell line | AU | | | AL | | |
| Time (30min) | | | | | | |
|  | MIC 1  (36 µg/mL) | MIC 2  (72 µg/mL) | MIC 3  (108 µg/mL) | MIC 1  (49 µg/mL) | MIC 2  (98 µg/mL) | MIC 3  (147 µg/mL) |
| Tonsillar | 82.3 | 89.5 | 82.6 | 87.3 | 91.9 | 97.8 |
| Buccal | 84.9 | 91.7 | 82.1 | 91.0 | 92.6 | 90.0 |
| FOM | 91.6 | 93.6 | 94.1 | 90.3 | 90.0 | 79.3 |
| Uvular | 77.5 | 77.5 | 87.6 | 87.0 | 75.8 | 97.7 |
| Gingival | 84.4 | 82.9 | 80.9 | 78.6 | 96.5 | 100 |
| Time (60min) | | | | | | |
| Tonsillar | 84.8 | 90.9 | 84.5 | 92.3 | 88.6 | 100 |
| Buccal | 87.9 | 86.2 | 92.0 | 85.3 | 88.2 | 95.6 |
| FOM | 92.4 | 93.1 | 92.1 | 81.7 | 86.1 | 94.0 |
| Uvular | 71.3 | 84.2 | 81.4 | 69.6 | 73.5 | 96.0 |
| Gingival | 82.9 | 84.1 | 75.6 | 86.8 | 100 | 100 |
| Time (120min) | | | | | | |
| Tonsillar | 87.2 | 89.6 | 89.3 | 94.8 | 100 | 100 |
| Buccal | 93.3 | 96.5 | 94.2 | 68.7 | 79.0 | 96.7 |
| FOM | 90.1 | 95.7 | 97.1 | 93.4 | 95.5 | 99.7 |
| Uvular | 91.0 | 95.5 | 96.0 | 81.4 | 91.0 | 100 |
| Gingival | 91.8 | 93.1 | 88.8 | 96.7 | 100 | 100 |

Green presenting ≥ 95% clearance and orange representing 90-94.9% clearance

# **Supplementary Table 4C**

| **Pre exposure clearance of FA1090 in oropharyngeal cells using AU and AL at 300µg/mL** | | |
| --- | --- | --- |
| Cell line | AU | AL |
| Time (30min) | | |
|  | MIC (300 µg/mL) | MIC (300 µg/mL) |
| Tonsillar | 73.3 | 97.4 |
| Buccal | 55.2 | 83.3 |
| FOM | 52.5 | 94.4 |
| Uvular | 93.3 | 97.4 |
| Gingival | 88.3 | 99.2 |
| Time (120min) | | |
| Cell line | MIC (300 µg/mL) | MIC (300 µg/mL) |
| Tonsillar | 94.3 | 98.7 |
| Buccal | 84.1 | 99.1 |
| FOM | 87.6 | 97.5 |
| Uvular | 98.3 | 97.4 |
| Gingival | 99.7 | 99.9 |

Green presenting ≥ 95% clearance and orange representing 90-94.9% clearance

# **Supplementary Figure 1**

Supplementary Figure 1: Representative micrographs of oropharyngeal cells. Images (FLoid cell imaging system, Life Technologies) with magnification at 80x. Scale bar of 125µm applies to all panels.

# **Supplementary Figure 2**

Supplementary Figure 2: Isolation and Immortalization of Primary Human Oropharyngeal Cells (Uvular Tissue): Panels A–E show representative images confirming successful immortalization of primary oropharyngeal keratinocytes using a doxycycline-inducible EGFP lentiviral system. Cells were immortalized with SV40 virus and subsequently transduced with an EGFP-expressing virus. EGFP expression was induced with 0.1% doxycycline, while the control group received no doxycycline. GFP fluorescence confirmed successful gene expression. Images were captured using the Invitrogen™ FLoid™ Cell Imaging Station. Bright-field images were taken at 80× magnification with 21% brightness; corresponding green fluorescence images were acquired at 80× magnification with 40% brightness.

# **Supplementary Figure 3**

Supplementary Figure 3: PCR detection of SV40 expression in immortalized uvula cells. PCR amplification of the SV40 large T antigen was performed using genomic DNA from uvula-derived epithelial cells at different passages. PCR products were separated on a 1% agarose gel in 1X TAE buffer and visualized under UV illumination. Lane M: DNA ladder; Lane P1: passage 10 cells; Lane P2: passage 25 cells; Lane C: positive control (SV40 viral DNA).
